# Fluid therapy in mechanically ventilated critically ill children: the sodium, chloride and water burden of fluid creep

**DOI:** 10.1186/s12887-020-02322-3

**Published:** 2020-09-05

**Authors:** Thomas Langer, Veronica D’Oria, Giulia C. I. Spolidoro, Giovanna Chidini, Stefano Scalia Catenacci, Tiziana Marchesi, Marta Guerrini, Andrea Cislaghi, Carlo Agostoni, Antonio Pesenti, Edoardo Calderini

**Affiliations:** 1 Department of Medicine and Surgery, University of Milan-Bicocca, Milan, Italy; 2 Fondazione IRCCS Ca’ Granda Ospedale Maggiore Policlinico, Anestesia e Terapia Intensiva Donna-Bambino, Milan, Italy; 3 Department of Pathophysiology and Transplantation, University of Milan, Milan, Italy; 4 Department of Clinical Sciences and Community Health, University of Milan, 20122 Milan, Italy; 5 Fondazione IRCCS Ca’ Granda Ospedale Maggiore Policlinico, Pediatric Intermediate Care Unit, 20122 Milan, Italy; 6 Department of Anesthesia, Critical Care and Emergency, Fondazione IRCCS Ca’ Granda Ospedale Maggiore Policlinico, Milan, Italy

**Keywords:** Fluid overload, Maintenance fluids, Hyperchloremia, Fluid therapy, Intensive care units, pediatric, Sodium, Chloride, Water-electrolyte balance

## Abstract

**Background:**

Fluid therapy is a cornerstone of pediatric intensive care medicine. We aimed at quantifying the load of water, sodium and chloride due to different fluid indications in our pediatric intensive care unit (PICU). We were particularly interested in the role of fluid *creep*, i.e. fluid administered mainly as the vehicle for drugs, and the association between sodium load and water balance.

**Methods:**

Critically ill children aged ≤3 years and invasively ventilated for ≥48 h between 2016 and 2019 in a single tertiary center PICU were retrospectively enrolled. Need for renal replacement therapy, plasmapheresis or parenteral nutrition constituted exclusion criteria. Quantity, quality and indication of fluids administered intravenously or enterally, urinary output and fluid balance were recorded for the first 48 h following intubation. Concentrations of sodium and chloride provided by the manufacturers were used to compute the electrolyte load.

**Results:**

Forty-three patients (median 7 months (IQR 3–15)) were enrolled. Patients received 1004 ± 284 ml of water daily (153 ± 36 ml/kg/day), mainly due to enteral (39%), *creep* (34%) and maintenance (24%) fluids. Patients received 14.4 ± 4.8 mEq/kg/day of sodium and 13.6 ± 4.7 mEq/kg/day of chloride, respectively. The majority of sodium and chloride derived from fluid *creep* (56 and 58%). Daily fluid balance was 417 ± 221 ml (64 ± 30 ml/kg/day) and was associated with total sodium intake (r^2^ = 0.49, *p* < 0.001).

**Conclusions:**

Critically ill children are exposed, especially in the acute phase, to extremely high loads of water, sodium and chloride, possibly contributing to edema development. Fluid *creep* is quantitatively the most relevant fluid in the PICU and future research efforts should address this topic in order to reduce the inadvertent water and electrolyte burden and improve the quality of care of critically ill children.

## Background

Water is the main constituent of the human body. Indeed, 60–70% of the body weight of an adult is made of water and the percentage rises up to 75–80% when dealing with infants and young children [1–3]. Due to several reasons, including this very high total body water content, greater insensible losses caused by a higher surface area to body mass ratio [4], a higher metabolic rate [5] and the possible presence of immature regulatory mechanisms [6, 7], children (especially newborns and children up to three years) develop frequent water and electrolyte imbalances.

Intravenous fluid therapy is therefore a cornerstone of pediatric critical care medicine and the prescription of intravenous fluids needs to be approached as any other pharmacological treatment, i.e., intravenous fluids have clear indications, contraindications and possible side effects [8]. Classically, only three clinical indications for intravenous fluid prescription exist:

1) Fluid resuscitation, i.e. rapid infusion of parenteral fluids in order to correct an acute intravascular fluid deficit [9]; 2) fluid replacement, i.e. parenteral fluids administered in stable patients in order to replace past or ongoing extracellular fluid losses, when oral fluid intake is inadequate, and 3) fluid maintenance, i.e. to provide water and electrolytes in hemodynamically stable children that are not able/allowed to drink water [10].

In addition, a substantial and frequently neglected water and electrolyte amount is administered to patients as a vehicle for intravenous drugs (sedatives, antibiotics, etc.), to provide patency of indwelling vascular catheters and to flush venous lines after blood withdrawal or drug administration [11–13]. This frequently neglected category of fluids has been recently termed as fluid *creep* [14], in analogy to the excess water administered inadvertently during burn care resuscitation [15].

Isotonic saline, i.e. NaCl 0.9%, which is constituted of 154 mEq of sodium and 154 mEq of chloride per Liter, is the classical fluid *creep* [13]. Besides the well-known side-effects of NaCl 0.9%, including hyperchloremic metabolic acidosis and hypernatremia [16–20], NaCl 0.9% contributes significantly to the total daily intake of sodium and chloride. It is important to underline the fact that recommended sodium intakes for both adults and children range between 1 and 2 mEq/kg/day [21]. Furthermore, it is well known that during an abrupt increase in sodium intake, in healthy subjects, only a fraction of the excessive sodium is excreted on the first day, leading to a positive sodium balance, thus increasing the body’s sodium stores and favoring a consensual water retention and weight gain [22]. It is conceivable that these mechanisms are even more pronounced in critically ill patients who frequently have altered homeostatic mechanisms. This, of course, might foster the formation of edema and favor the worsening of the clinical condition.

The aim of the present retrospective study is to describe the current clinical practice regarding fluid therapy in the pediatric intensive care unit (PICU) of a tertiary Italian referral hospital, quantify the load of water, sodium and chloride due to different fluid indications and assess the association between sodium intake and water balance.

## Methods

This retrospective, single center study was approved by the institutional review board of our hospital (decision number 502/2019). The need for informed consent was waived owing to the retrospective nature of the study.

All patients admitted to the 5-bed tertiary PICU of the Fondazione IRCCS Ca′ Granda – Ospedale Maggiore Policlinico, Milan between January 2016 and the end of May 2019 were screened for eligibility. The inclusion criteria were age ≤ 3 years and ≥ 48 h of continuative invasive mechanical ventilation. Exclusion criteria included patients < 28 days, patients who received renal replacement therapy, plasmapheresis and parental nutrition. Patients were studied for 48 h starting from the time of intubation or PICU admission if the endotracheal tube was already in place.

Clinical data were extracted from the patient data management system of our PICU (Digistat ICU- Ascom software, Scandicci, Italy). Demographic data included age, gender, body weight, height, presence and type of comorbidities. The cause of PICU admission was categorized in 4 classes: respiratory failure, sepsis/septic shock, neurological disease and post-operative. Several variables were extracted from the digital records in order to describe the severity of patients’ clinical condition. These included the use of vasopressors and/or inotropes (epinephrine, norepinephrine, dopamine or dobutamine), rescue respiratory strategies (high frequency oscillatory ventilation (HFOV), prone position) and worst values of the 48-h study period for positive end-expiratory pressure (or mean airway pressure in case of HFOV), fraction of inspired oxygen (FiO_2_), and ratio of arterial partial pressure of oxygen to FiO_2_ (P/F ratio).

In addition, data regarding administered intravenous fluids were collected. Of note, our patient data management system records semi-automatically, on an hourly basis, the type and amount of parenteral fluids administered through infusion/peristaltic pumps, while the nurses input in the system the amount and type of any other fluid administered manually. Furthermore, the patient data management system records the type and amount of enteral nutrition and/or enteral water administered to the patients.

The amount of sodium and chloride administered with each fluid was calculated multiplying the amount of intravenous fluid by the electrolyte concentration declared by the manufacturer or as assessed in previous studies [17]. In addition, the amount of sodium administered with enteral nutrition was calculated considering the electrolyte composition of the enteral preparations, as declared by the manufacturer and as summarized in Table 1.

**Table 1:** Composition of enteral formulae employed in the study population

Preparation	H_2_O [ml]	Na [mg]	Na [mEq]	Cl [mg]	Cl [mEq]
Human milk	87.7	23.0	1.0	46.0	1.3
Nidina 1	87.0	23.4	1.0	40.0	1.1
Nidina 2	86.4	26.0	1.1	49.0	1.4
Humana 1	86.0	20.0	0.9	45.0	1.3
Infatrini	85.0	37.0	1.6	62.0	1.8
Nutrini	85.0	60.0	2.6	95.0	2.7
Neocate	86.2	26.1	1.1	53.3	1.5
Aptamil proExpert	86.3	20.0	0.9	41.0	1.2
Nutramigen 2 LGG	84.4	21.0	0.9	47.0	1.3

Data refer to 100 ml of enteral formula. For powder preparations the standard dilution suggested by the manufacturer was applied. Data regarding human milk are an average value derived from the literature. Nidina 1 and Nidina 2 are produced by Nestlé Italia, Assago (MI), Italy; Humana 1 by Humana Italia, Milano, Italy; Infatrini, Nutrini and Neocate are produced by Nutricia Italia, Milano, Italy; Aptamil proExpert is produced by Mellin, Milano, Italy; Nutramigen 2 LGG is produced by Mead Johnson Nutrition, Rome, Italy

According to the medical records, each fluid administered was assigned to a fluid indication [14]: fluid resuscitation, maintenance/replacement, enteral nutrition, blood products, and fluid *creep*. Fluid creep was defined as the amount of fluid administered to patients as a vehicle for intravenous drugs (sedatives, antibiotics, etc.), to provide patency of indwelling vascular catheters and to flush venous lines after blood withdrawal or drug administration.

Daily forms included also urinary output and a simplified daily fluid balance, calculated by subtracting total urinary output from the total input of parenteral and enteral water. Finally, the percentage of water, sodium and chloride administered with each indication were computed.

### Statistical analysis

All data were tested for homogeneity of variance and normality of distribution using the Shapiro-Wilk test. Normally distributed data were expressed as mean ± standard deviation, while non-normally distributed data were reported as median and interquartile range. Pearson’s correlation was used to assess the association between sodium intake and cumulative water balance. Analysis was performed with SAS 9.4 (SAS Institute Inc., Cary, NC, USA) and Sigma Plot 12.0 (Systat Software Inc., San Jose, CA). A *P*-value lower than 0.05 was considered as statistically significant.

## Results

**Fig. 1:**
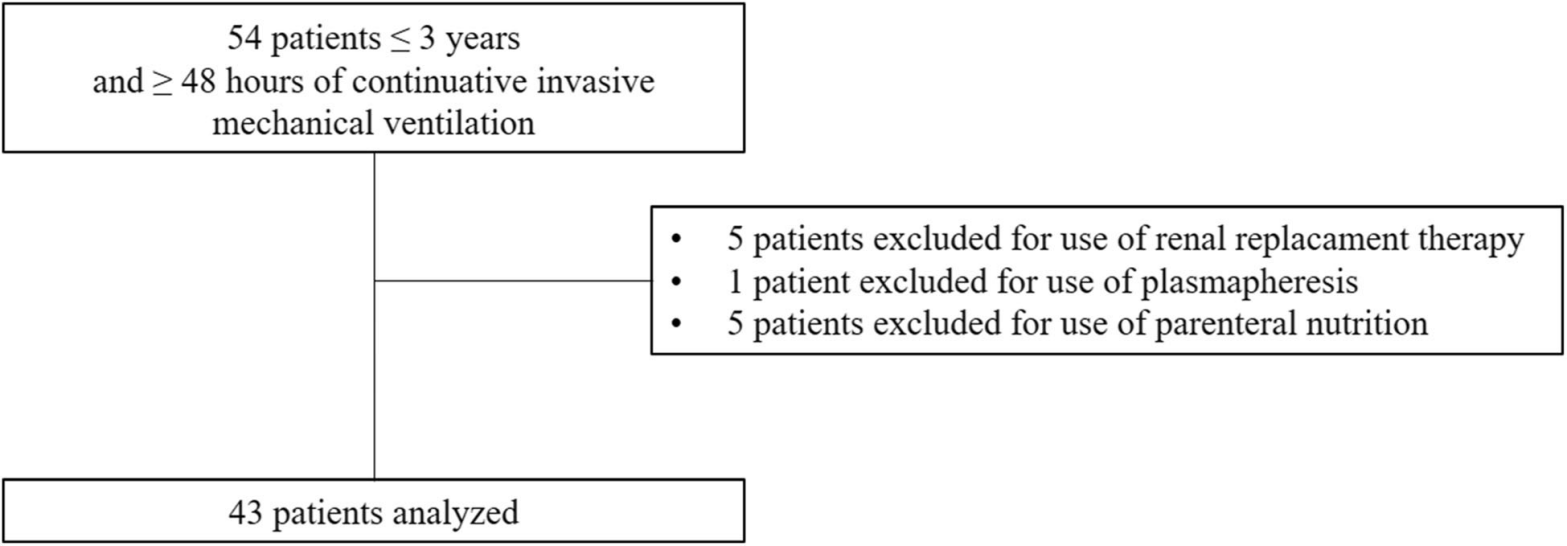
Study flow chart

Fifty-four patients fulfilled the inclusion criteria, 11 presented exclusion criteria, leaving 43 patients for the analysis (Fig. 1). Demographic and clinical characteristics of the study population are summarized in Table 2. Patients had a median age and weight of 7 [3–15] months and 6.6 [4.7–9.0] kg, respectively. Acute respiratory failure was the cause of PICU admission in 77% percent of included patients. Comorbidities were present in 51% of patients, including children with a history of premature birth (< 37 weeks of gestational age).

**Table 2:** Demographic and clinical data of the study population

Characteristics	(*n* = 43)
**Age, months**	7 [3–15]
**Weight, kg**	6.6 [4.7–9.0]
**Height, cm**	64 [55–74]
**Male – no. (%)**	23 (54)
**Comorbidities – no. (%)**	22 (51)
**Chronic lung disease – no. (%)**	2 (5)
**Oncological disease – no. (%)**	1 (2)
**Neurologic disorder – no. (%)**	10 (23)
**Ex-preterm – no. (%)**	9 (21)
**Cause of PICU admission – no. (%)**	
**Acute respiratory failure**	33 (77)
**Neurological disease**	3 (7)
**Post-operative**	3 (7)
**Sepsis/septic shock**	4 (9)
**PEEP, cmH**_**2**_**O**	10 [8–13]
**FiO**_**2**_	0.6 [0.5–0.8]
**P/F, mmHg**	100 [78–145] (*n* = 37)
**HFOV, no. (%)**	9 (21)
**Prone position, no. (%)**	24 (56)
**Vasopressors and inotrpes, no. (%)**	13 (30)
**PICU LOS, days**	9 [8–14]
**PICU mortality, no. (%)**	3 (7)

Demographic and clinical data of the study cohort. Data are expressed as median [interquartile range] or as number (percentage). Reported respiratory variables refer to the worst values of the 48-h study period. PEEP = positive end-expiratory pressure; FiO_2_ = Fraction of inspired oxygen; P/F = ratio between arterial partial pressure of oxygen and FiO_2_; HFOV = high frequency oscillatory ventilation; Vasopressors and inotropes = use of vasopressors or inotropes (epinephrine, norepinephrine, dopamine or dobutamine) during the study period; PICU LOS = PICU length of stay

Acetated Ringer’s was the preferred choice for both fluid resuscitation (88 ± 33%) and fluid maintenance (88 ± 26%). The other employed intravenous fluid for these purposes was NaCl 0.9%, which was also the only type of fluid used for fluid *creep*. Table 3 summarizes total daily amount of water, sodium and chloride administered during the study period. On average, a daily dose of 1004 ± 284 ml (153 ± 36 ml/kg/day) of water was administered. The major source of water input was enteral nutrition (60 ± 23 ml/kg/day, 39% of total water input), followed by fluid *creep* (51 ± 23 ml/kg/day, 34.2%) and fluid maintenance (34 [21–50] ml/kg/day, 23.4%). The quantitative role of fluid boluses/fluid resuscitation was minor (1.7%) and quantitatively similar to blood components (1.5%).

**Table 3:** Daily Water, Sodium, Chloride input, and source

Variables	Water [ml/kg/day]	Sodium [mEq/kg/day]	Chloride [mEq/kg/day]
Input	153 ± 36	14.4 ± 4.8	13.6 ± 4.7
Resuscitation fluids	0 [0–5]	0.0 [0.0–0.7]	0.0 [0.0–0.6]
Maintenance fluids	34 [21–50]	4.5 [2.7–6.7]	3.7 [2.3–5.6]
Fluid creep	51 ± 23	7.9 ± 3.6	7.9 ± 3.6
Enteral water	60 ± 23	1.0 ± 0.4	1.0 ± 0.5
Blood components	0 [0–6]	0.0 [0.0–0.8]	0.0 [0.0–0.6]

Data regarding water refer to milliliters per kg per day, while data on sodium and chloride refer to milliequivalents per kg per day. Data are expressed as mean (standard deviation) or median (interquartile range) according to their distribution

Fluids administered as a vehicle for intravenous drugs constituted 76 ± 12% of fluid creep, while fluids administered to provide patency of catheters and for flushes constituted the remaining 24 ± 12% of fluid creep. Among intravenously administered drugs, sedatives and antibiotics where the two major sources of fluid creep (44 ± 22% and 18 ± 18%, respectively).

The daily dose of sodium was 97 ± 42 mEq (14.4 ± 4.8 mEq/kg/day). Fluid creep represented the major source of sodium (7.9 ± 3.6 mEq/kg/day, 55.7% of total sodium input), followed by maintenance fluids (32.8%), enteral feed (6.9%), fluid resuscitation (2.3%) and blood components (2.2%) (Table 3).

**Fig. 2:**
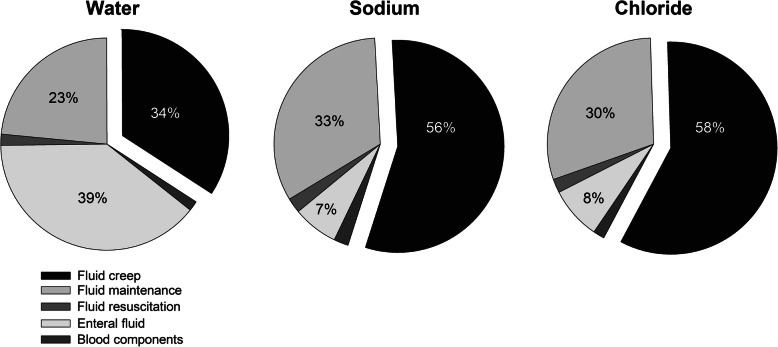
The three pie charts represent the percentage contribution of each fluid category to water, sodium and chloride input. Percentages are reported numerically within the slice for Fluid Creep, Enteral Fluid and Maintenance fluids. Both fluid resuscitation and blood components contributed approximately 2% to water, sodium and chloride input. The “exploded” pie slice refers to Fluid Creep

The daily dose of chloride was 92 ± 40 mEq (13.6 ± 4.7 mEq/kg/day), slightly lower than sodium. Fluid creep represented the major source of chloride (7.9 ± 3.6, 58.2% of total chloride input), followed by maintenance fluids (29.9%), enteral feed (8%), fluid resuscitation (2.1%) and blood components (1.7%). Figure 2 represents the pie charts summarizing the percentage contribution of different fluid categories to water, sodium and chloride load.

**Fig. 3:**
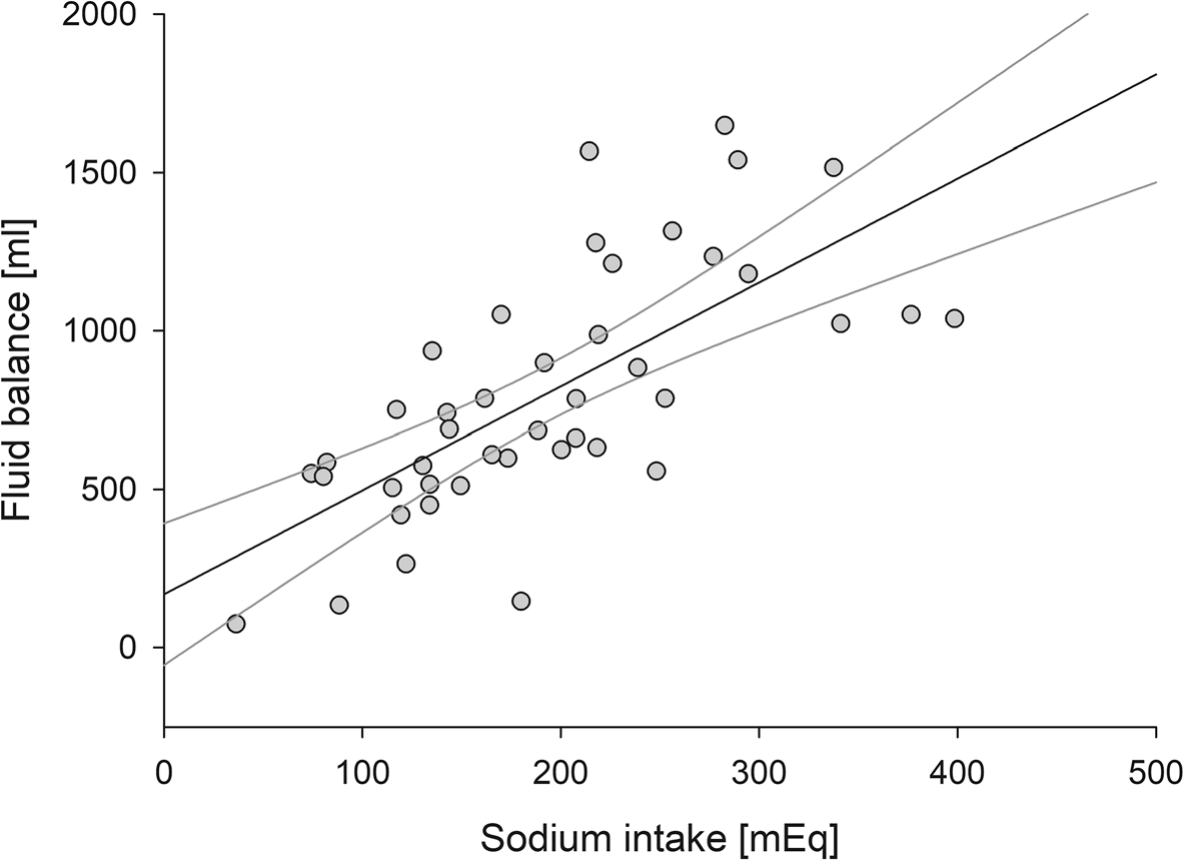
Correlation between total sodium intake and cumulative 48-h fluid balance. Fluid balance was calculated as total fluid intake (parenteral and enteral) – urinary output. The regression line (y = 168 + 3.3 x; r^2^ = 0.49, *p* < 0.0001) and 95% confidence band are reported

Patients had an average daily urinary output of 602 ± 242 ml, i.e. 92 ± 32 ml/kg per day. Given the total fluid input (Table 3), the result was an average daily positive fluid balance of 417 ± 221 ml, i.e. 64 ± 30 ml/kg per day. Figure 3 represents the association between the total 48-h sodium intake and total fluid balance. As can be noted, the total amount of sodium administered was strongly associated (r^2^ = 0.49, *p* < 0.0001) with cumulative fluid balance.

## Discussion

We studied a population of young (≤3 years), severely ill, mechanically ventilated children during the first 48 h of the acute phase. The main findings of our study are (i) the overall extremely high water, sodium and chloride load, (ii) the predominant role of fluid *creep*, especially regarding electrolytes’ input, and (iii) the strong association between total sodium input and positive fluid balance.

In our population, the major source of water input was enteral nutrition, which already provided approximately 65% of expected water needs for healthy children, according to Holliday and Segar [23]. The second water source was fluid *creep*, accounting for 34% of water input, followed by fluid maintenance. As already pointed out by other authors [14, 24], also in our patients the quantitative role of fluid boluses/fluid resuscitation and blood components was minor. Given the quantitative amount and the composition of employed fluid *creep*, it is not surprising that this category of fluids represented the major source of sodium (56% of total sodium input, Fig. 2). Overall, the sodium load exceeded approximately 10 times the recommended sodium input [21]. Of course, it is likely that the recommended sodium inputs might not be sufficient for critically ill patients with altered vascular permeability, i.e. in patients with relative hypovolemia. Nevertheless, the administration of 56% of this load was inadvertent, i.e., from fluids administered as vehicle for intravenous drugs and to guarantee patency of indwelling intravascular catheters, namely fluid *creep*. It is therefore conceivable that this sodium load was not a therapeutic choice, but rather a side effect of other therapies. It might be interesting underling the fact that the sodium load deriving from enteral preparations was, on average, 1.0 ± 0.4 mEq/kg/day, i.e. very close to the recommended sodium input. This is reasonable, as enteral feeds have been prepared in order to cover all the nutritional needs, including electrolytes.

The total chloride load was slightly lower than sodium (13.6 ± 4.7 mEq/kg/day). The prevalent use of acetated Ringer’s as maintenance fluid explains this finding. Indeed, acetated Ringer’s has a sodium and chloride concentration of 132 and 110 mEq/L, respectively. Consequently, the chloride load deriving from this source is slightly lower than the corresponding sodium load. On the contrary, sodium and chloride load deriving from fluid *creep* are equal (7.9 ± 3.6 mEq/kg/day) as NaCl 0.9% was used for this purpose.

The high chloride burden due to fluid therapy in general, and fluid *creep* in particular, might contribute to the development of hyperchloremia, with the related side effects [25]. Hyperchloremia might cause metabolic acidosis [16, 20], alter the contractility of arteriolar smooth muscles [26, 27], potentially altering renal perfusion and contributing to renal dysfunction [28]. Furthermore, high chloride concentrations might favor lower urinary sodium excretion thus contributing to edema formation [29, 30]. Indeed, despite a preserved renal function, the fluid balance was extremely positive (64 ± 30 ml/kg/day), significantly higher than the one reported in previous studies [11]. This is likely due to the fact that we selected a population of young, particularly sick and mechanically ventilated children. These three factors taken together certainly amplified the clinical significance. Nevertheless, fluid overload, a major issue in the ICU, is associated with worse clinical outcomes [31]. Interestingly, a good linear correlation was found between total sodium intake and fluid balance (Fig. 3). Again, it is worth repeating that 56% of the sodium load was inadvertent, i.e. it derived from fluid *creep*. When analyzing the two major components of fluid creep, we found that the majority (76%) was due to the dilution of intravenous drugs, while the remaining part (24%) was due to the use of fluids to guarantee catheters’ patency.

The cultural awareness regarding intravenous fluids is certainly increasing [32–35] and careful determination is occurring with regards to the indication, the preferred composition and the possible side effects to intravenous fluids. However, most of the scientific attention has been directed towards resuscitation, replacement and maintenance fluids.

Indeed, there is strong consensus regarding the tonicity of fluids for resuscitation and replacement, i.e., in this context isotonic, possibly balanced fluids should be preferred. On the contrary, there is a great debate regarding the appropriate tonicity of maintenance fluids [36–39]. Many clinicians suggest to use isotonic maintenance fluids in order to avoid the risk of hyponatremia and cerebral edema [40]. On the contrary, other authors promote the use of hypotonic fluids in order to avoid “water and salt intoxication” [41, 42].

Finally, it is not surprising that, at the time of writing, no indication exists regarding fluid *creep*. Indeed, these fluids have been neglected for a very long time and only recently, the research interest in the topic is increasing [11, 12, 14, 24, 43, 44]. However, given the fact that fluid *creep* can account up to 50% of daily parenteral fluid administration and a similar percentage of total daily sodium load in critically ill children admitted to the PICU, we can firmly state that they are conceptually as important as, and quantitatively even more important than resuscitation, replacement and maintenance fluids. Indeed, in a recent before-and-after study performed in a single center adult ICU, Bihari and colleagues demonstrated that the sodium burden and positive fluid balance deriving from *fluid creep* can be safely reduced by switching from an isotonic solution, such as NaCl 0.9%, to a hypotonic solution, such as 5% glucose [45]. This important research topic needs to be addressed further, possibly with randomized control trials, also in the pediatric population.

Other possible strategies to reduce the water, sodium and chloride burden of intravenous fluids would be to favor, whenever possible, the enteral route for drug administration; to concentrate as much as possible parenteral drugs; to review, on a daily basis, the indications of intravenous fluids, i.e. to adopt strategies of fluid stewardship [46] and to avoid displaying tracings of central venous pressure or arterial pressure, unless strictly necessary. Indeed, pressure transducers used in our PICU require a continuous drip of 3 ml/h of fluid. If both an arterial and central line are in place, this already results in a daily load of 144 ml of water and 22 mEq of sodium and chloride.

There were several limitations to our study. First, the study was retrospective in nature and data derived from only 43 patients of the same PICU. Consequently, the small number of patients hampers the generalizability of our findings. The extensive use of non-invasive ventilation in our PICU and the established tendency to intubate and ventilate invasively patients only as last resort, might in part explain the limited number of included patients. Second, we had no data on urinary sodium and chloride excretion. It was thus impossible to assess sodium and chloride balance. Third, we did not consider the sodium content of antibiotics, which can be significant [12]. Finally, it is possible that some flushes or extemporary therapies were not inserted manually in the patient management system. Taking together these last two aspects, it is conceivable that the effective water, sodium and chloride load might have been slightly underestimated.

## Conclusions

In conclusion, our study described the clinical practice regarding fluid administration in critically ill, mechanically ventilated patients under 3 years of age, treated at our PICU. We had established protocols in our unit for drug dilution, not necessarily employing the maximal possible concentration. Furthermore, we did not have local protocols to reduce the inadvertent amount of fluids deriving from crystalloids used to guarantee the patency of catheters. As a result, our current clinical approaches and habits unequivocally lead to an inadvertent and excessive water, sodium and chloride load, which might contribute to the development of edema and significantly impact on patients’ outcomes. Fluid *creep* is quantitatively the most relevant fluid in the PICU and future research and clinical efforts should address this topic in order to improve the quality of care of critically ill children.

## Data Availability

All data generated or analysed during this study are made available from the authors.

## Abbreviations

Cl: Chloride
FiO_2_: Fraction of inspired oxygen
HFOV: High frequency oscillatory ventilation
IQR: Interquartile range
IRCCS: Istituto di Ricerca e Cura a Carattere Scientifico
Na: Sodium
P/F ratio: Ratio of arterial pressure of oxygen to FiO_2_
PEEP: Positive end-expiratory pressure
PICU: Pediatric Intensive Care Unit
PICU LOS: PICU length of stay
